# Secretion of beta-human chorionic gonadotropin by non-small cell lung cancer: a case report

**DOI:** 10.1186/1752-1947-5-19

**Published:** 2011-01-19

**Authors:** Saakshi Khattri, Abhirami Vivekanandarajah, Seema Varma, Frank Kong

**Affiliations:** 1 Department of Internal Medicine, Staten Island University Hospital, 475 Seaview Ave, Staten Island, New York 10305, USA; 2 Department of Hematology Oncology, Staten Island University Hospital, 475 Seaview Ave., Staten Island, New York 10305, USA; 3 Department of Pathology, Staten Island University Hospital, 475 Seaview Ave., Staten Island, New York 10305, USA

## Abstract

**Introduction:**

We describe a case of non-small cell lung cancer that was found to stain positive for beta-human chorionic gonadotropin on immunohistochemistry. Only a few case reports have described lung cancers that secrete beta-human chorionic gonadotropin.

**Case presentation:**

A 68-year-old Caucasian man presented with symptoms of weakness, fatigue and weight loss for the past two months. On examination, he was found to have generalized lymphadenopathy, and radiologic workup revealed numerous metastases in the lungs, liver and kidneys. Biopsy of the supraclavicular lymph node revealed metastatic large cell lung cancer with beta-human chorionic gonadotropin hormone positivity. The serum beta-human chorionic gonadotropin level was 11,286 mIU/ml (upper limit of normal, 0.5 mIU/ml in non-pregnant females). He was diagnosed with stage 4 lung non-small cell lung cancer. The patient refused chemotherapy. He was discharged home with hospice care.

**Conclusion:**

The markedly elevated serum values of beta-human chorionic gonadotropin initially prompted the medical team to investigate germinal tumors. In the presence of a negative testicular ultrasound, workup was performed to find an extratesticular source of the tumor. Finally, the diagnosis was made with a tissue biopsy. This case illustrates that atypical markers can be seen in many cancers, emphasizing the role of immunohistochemistry and tissue biopsy in establishing the diagnosis.

## Introduction

β-human chorionic gonadotropin (β-hCG) is commonly produced by germ cell tumors and seldom produced by other tumors. In the literature, a few case reports discuss the ectopic production of β-hCG in small cell and non-small cell lung cancers. We present an unusual case of lung cancer with ectopic production of β-hCG.

## Case presentation

A 68-year-old Caucasian male patient with medical history significant for depression, emphysema, and gastroesophageal reflux disease presented to his primary care physician for a routine office visit. Medications at home included paroxetine, tiotropium, and omeprazole. Blood work revealed a hemoglobin level of 7.4 mg/dl with a hematocrit of 20 mg/dl, and then he was sent to the Emergency Department for transfusion. He reported that he had experienced decreased appetite and significant weight loss for the past two months. He had never seen a primary doctor until recently, when he was diagnosed with depression, emphysema, and gastroesophageal reflux disease. Family history was significant for a sister with colon cancer and his mother with multiple myeloma. He was a long-term smoker with an 80-pack-year history of smoking. On physical examination, vital signs were BP, 112/68 mm Hg; RR, 16/minute; PR, 88/minute; and temperature, 97.9°F. Chest auscultation revealed diffusely scattered coarse rhonchi. The abdomen was soft with no organomegaly. The testes were soft and were not enlarged. No lymphadenopathy was noted.

**Figure 1:**
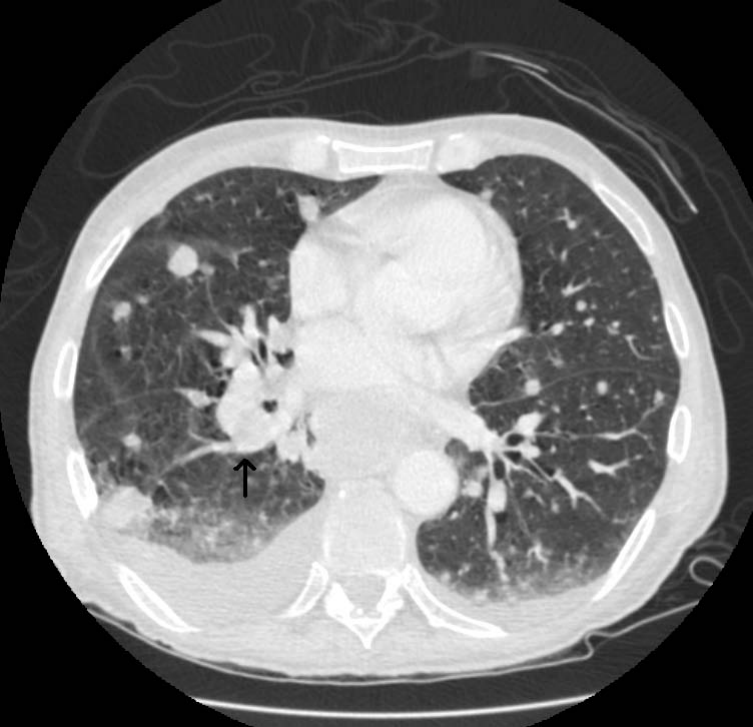
Right hilar mass noted with multiple pulmonary nodules scattered throughout the lung parenchyma.

**Figure 2:**
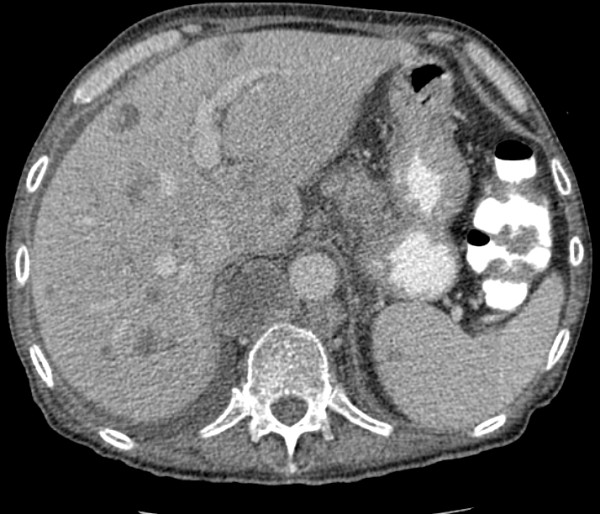
Multiple hypodensities noted in the liver, and an isolated lesion noted in the head of the spleen.

**Figure 3:**
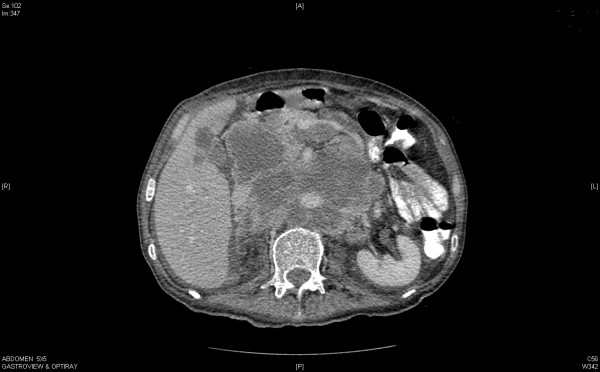
A massive conglomerate of periaortic, aortocaval lymph nodes and retroperitoneal necrotic mass measuring up to 14.4 cm, which encases the abdominal vasculature.

**Figure 4:**
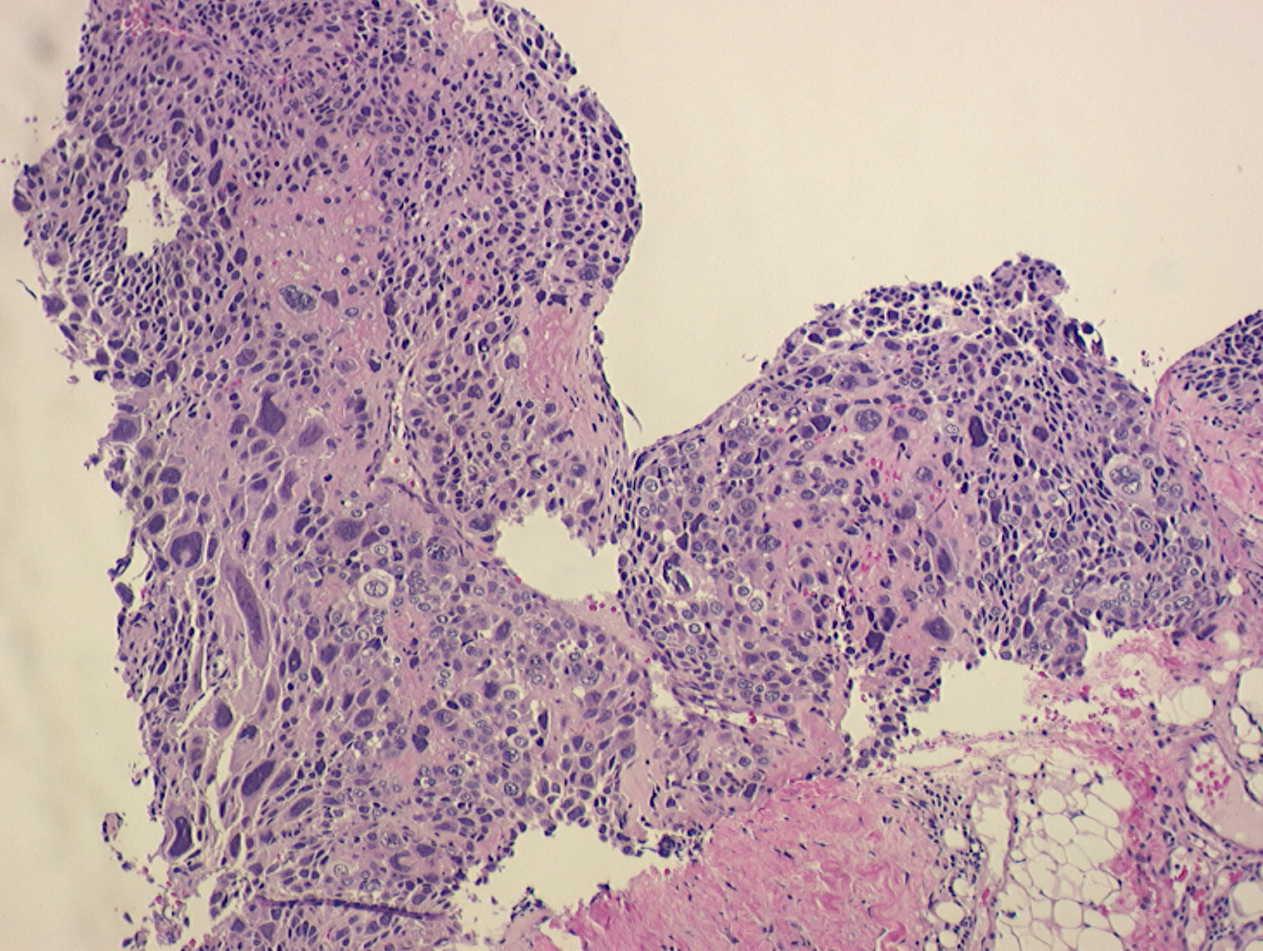
Biopsy of supraclavicular lymph node showing undifferentiated giant cells.

**Figure 5:**
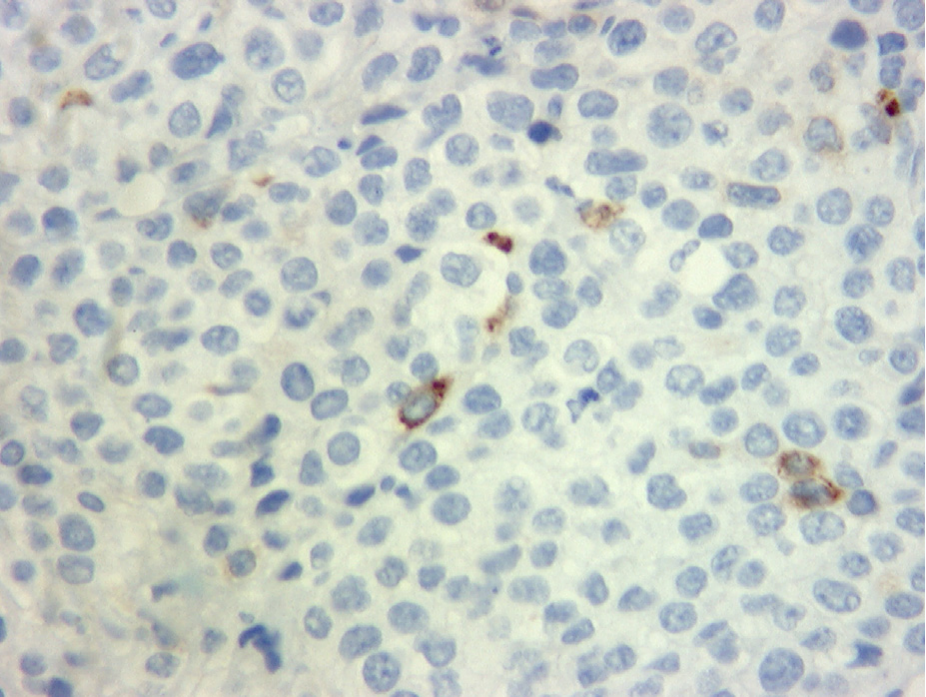
Immunohistochemistry showing focal positivity for β-hCG.

**Figure 6:**
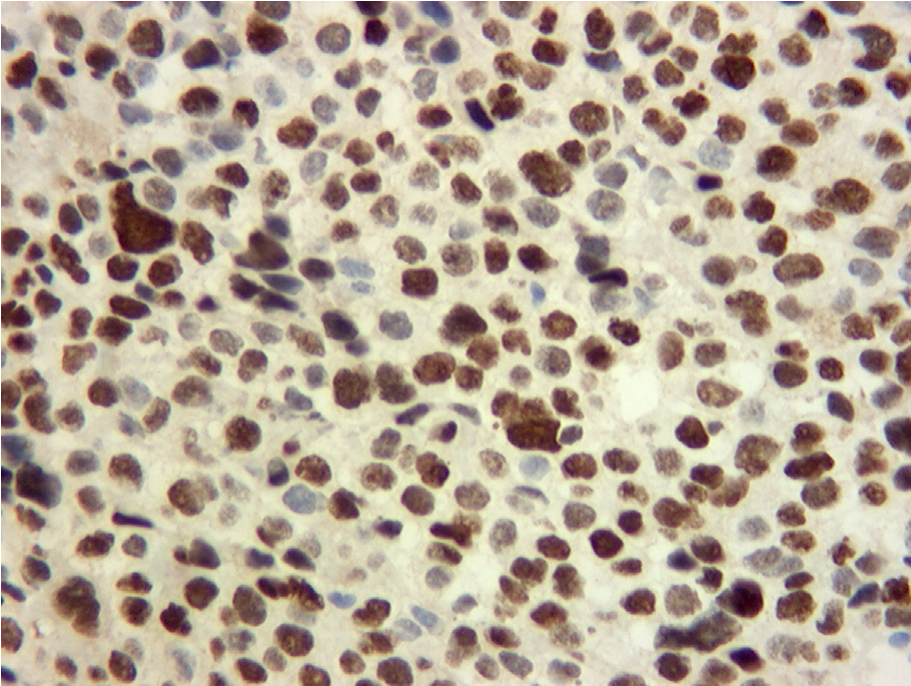
Immunohistochemistry showing cells that stain positive for P63, highly suggestive of squamous cell carcinoma.

**Figure 7:**
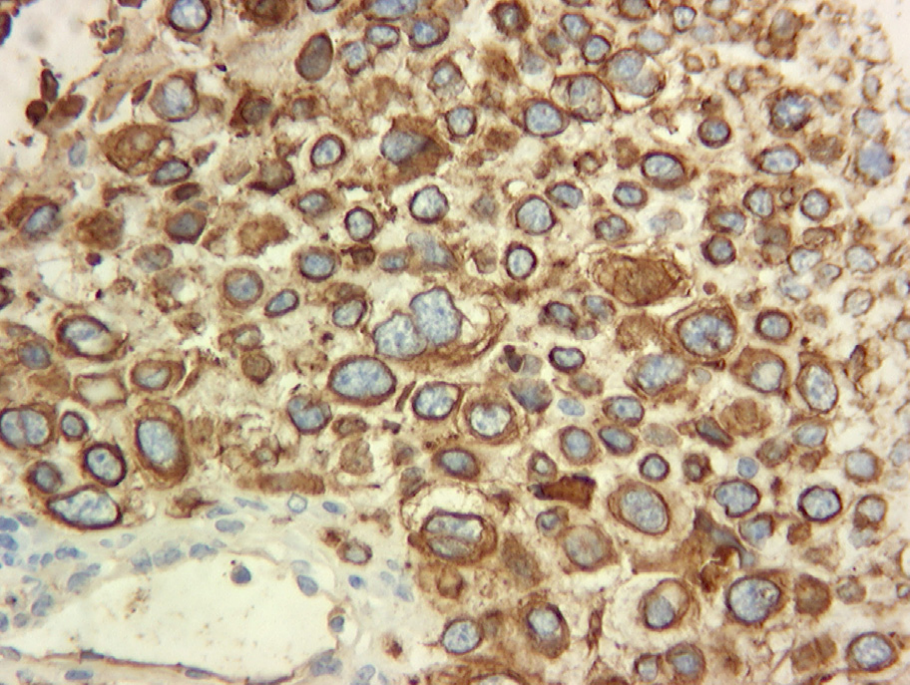
Immunohistochemistry showing positivity for CK 7 on the cell membrane, suggestive of a carcinoma, most likely lung.

In the context of the anemia and the recent weight loss, a workup for malignancy was initiated. The patient underwent colonoscopy and esophagogastroduodenoscopy (EGD). No polyps or ulcerated lesions were noted on the colonoscopy. The EGD revealed esophageal candidiasis and chronic gastritis. Computed tomography scans of the chest, abdomen, and pelvis revealed extensive generalized lymphadenopathy. The left supraclavicular, paraesophageal, paratracheal, and subcarinal lymph nodes were enlarged and a 2.3 cm right hilar mass was seen. Multiple nodules were found in the lungs bilaterally, the largest one measuring 2.7 cm in diameter. A 2.5 cm mass was noted in the periphery of the left upper lobe (Figure 1). Several hypodensities were noted in the kidneys, liver, and spleen (Figure 2). An ill-defined necrotic retroperitoneal mass measuring 14.4 cm, encasing the abdominal vasculature, was seen in the periaortic and aortocaval areas (Figure 3). At that point, the working diagnosis was metastasis with an unknown primary tumor. Differential diagnoses included lung cancer and germ cell tumors. Further blood work revealed a β-hCG level of 11,286 mIU/ml. α-Fetoprotein and prostate-specific antigen were negative. Ultrasound of the testes revealed neither testicular enlargement nor lesions. At that point, the possibility of primary testicular germ cell tumor was excluded. On day 4, the patient underwent a left supraclavicular lymph node excision. The histopathology revealed metastatic poorly differentiated squamous cell carcinoma with focal positivity for β-hCG (Figures 4 and 5). Immunohistochemistry revealed CK, 7; AE1/AE3, β-hCG, CAM 5.2, and P63 positivity (Figure 6). CK 20, CEA, CA 19-9 AFP, and TTF were negative (Figure 7). These markers were consistent with a poorly differentiated or undifferentiated non-small cell carcinoma (squamous type) with β-hCG positivity. He was diagnosed with stage 4 lung cancer with ectopic secretion of β-HCG. The patient and the family opted for palliative treatment.

## Conclusion

Lung cancer is the most common cause of worldwide cancer mortality in men and women, causing approximately 1.2 million deaths per year [1].

Ectopic beta human chorionic gonadotropin (β-hCG) expression by non-gestational tumors was noted in the early 1900s, and β-hCG secretion has been noted in gastric, ovarian, liver, and lung cancers [2]. Despite this, lung cancer with β-hCG production is rare, and only a few case reports have been published in the literature [3-5].

We do not know why non-gestational cells produce β-hCG. Several studies have tried to look into β-hCG production. In a study published from Japan, mRNA transcripts of the beta gene were detected in lung cancer tissues, and the result of the study was that β-hCG production was noted in malignantly transformed lung cells [6].

How β-hCG acts also is not clear. Some studies have indicated that it acts as an autocrine or paracrine growth factor or both by inhibiting apoptosis [7]. *In vitro *studies suggest that β-hCG may inhibit transforming growth factor beta (TGF-β) receptor complex by binding to a component of the receptor complex, thus blocking its binding sites. This prevents further interactions with other receptor components, eventually leading to apoptosis [7]. This may explain why β-hCG-producing tumors appear to be more aggressive and have a worse prognosis.

Similarly, other studies have shown that small cell lung cancer with β-hCG production results in a more-resistant tumor and worse prognosis, in chemoresistance, and that elevated β-hCG values are more commonly seen in patients with metastatic disease [8,9].

Although ectopic expression of β-hCG is now a recognized phenomenon, and studies have shown that its production by non-gestational tumors indicates a poorer prognosis, it is not clear whether it should be widely used as a prognostic marker and routinely measured in the patient's serum.

The mechanism of β-hCG production by the tumor cells is not well understood, and the action is at best speculative. Only a few studies have been done in this field, and more is required before it can be stated definitely that lung cancer with ectopic β-hCG production is indeed associated with a worse prognosis, worse stage, and chemoresistance.

## Abbreviations

AFP: α-fetoprotein; β-hCG: beta human chorionic gonadotropin; CA: carbohydrate antigen; CAM: anti-cytokeratin; CEA: carcinoembryonic antigen; CK: cytokeratin; EGD: esophagogastroduodenoscopy; TGF-β: transforming growth factor-beta; TTF-1: thyroid transcription factor 1.

## Consent

Written informed consent was obtained from the patient for publication of this case report and any accompanying images. A copy of the written consent is available for review by the Editor-in-Chief of this journal.

## Competing interests

The authors declare that they have no competing interests.

## Authors' contributions

SK, AV, and SV were the doctors taking care of the patient; SK and AV were responsible for analyzing the data. FK was responsible for the histologic examination. All authors read and approved the final manuscript.
